# Rat-to-Human Transmission of Cowpox Infection

**DOI:** 10.3201/eid0812.020089

**Published:** 2002-12

**Authors:** Tom F.W. Wolfs, Jaap A. Wagenaar, Hubert G.M. Niesters, Albert D.M.E. Osterhaus

**Affiliations:** * Wilhelmina Children’s Hospital/University Medical Center, Utrecht, the Netherlands; † Utrecht University, Utrecht, the Netherlands; ‡ University Hospital, Rotterdam, the Netherlands

**Keywords:** Cowpox, transmission, zoonosis

## Abstract

We isolated *Cowpox virus* (CPXV) from the ulcerative eyelid lesions of a 14-year-old girl, who had cared for a clinically ill wild rat that later died. CPXV isolated from the rat (*Rattus norvegicus*) showed complete homology with the girl’s virus. Our case is the first proven rat-to-human transmission of cowpox.

A 14-year-old girl was admitted to the hospital with ulcerated nodules on her upper lip and her left lower eyelid and several molluscum-like-lesions on her right eyelids (Figure 1). She was feverish but otherwise in good health. Values of complete blood and differential counts were within normal limits. The erythrocyte sedimentation rate was 41 mm the first hour, and she was treated orally with ciprofloxacin. Within a few days the lesions on her eyelids developed into crater-like ulcers, which were surrounded by inflammatory tissue and were later covered by thick black crusts; and sedema and erythema developed on the right side of her face. At home she kept turtles, hamsters, guinea pigs, birds, ducks, cats, and a dog. She also cared for a clinically ill wild rat, which she had found 2 weeks before admission. After 6 days of care, the rat died and was buried. During the following 4 weeks, the ulcerated lesions on the girl’s face healed and left atrophic scars.

**Figure 1:**
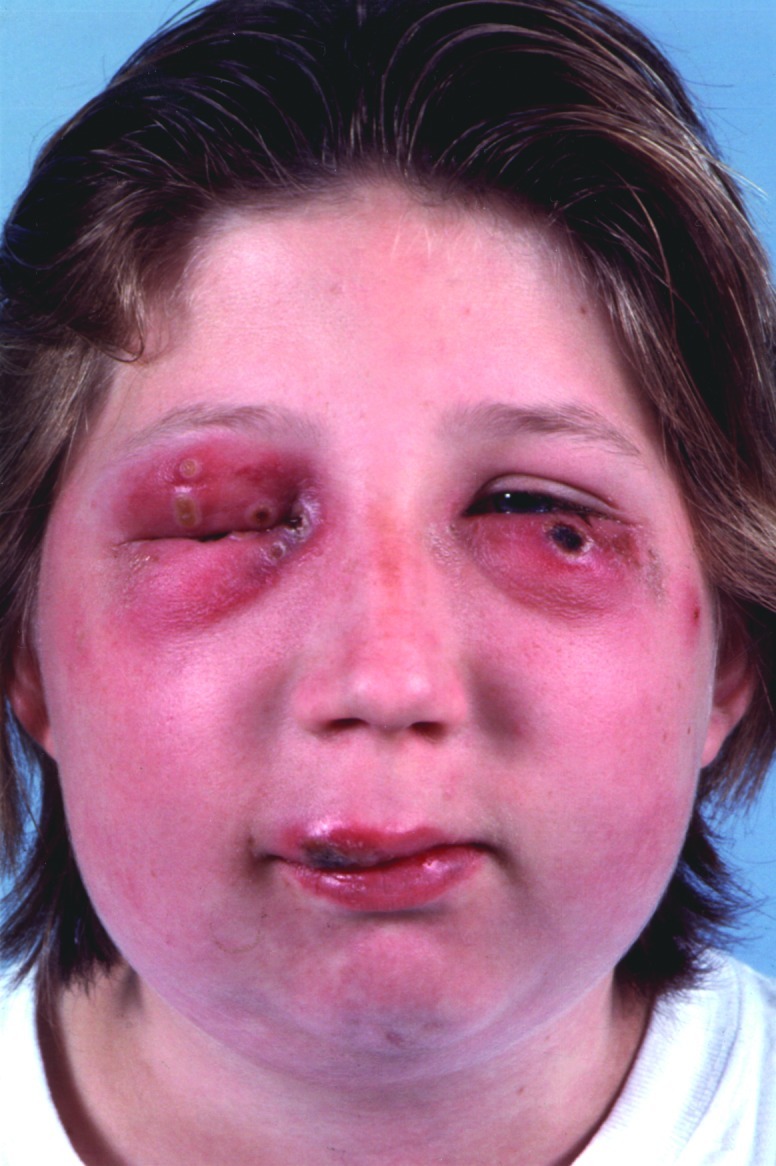
Cowpox lesions with ulcerated nodules on upper lip and left lower eyelid, several molluscum-like-lesions on the right eyelids, and oedema and erythema of the right side of the face.

Routine virus isolation procedures from biopsies of the eyelid lesions in Vero cells showed the presence of *Orthopoxvirus*. Serum immunoglobulin (Ig) M antibodies to the viral isolate and to *Vaccinia virus* were detected upon admission. The virus was identified as *Cowpox* *virus* (CPXV) by polymerase chain reaction (PCR) and sequence analysis (PCR directed at the *Orthopoxvirus* fusion protein gene, as described by Chantrey et al.) (1). The rat, identified as *Rattus norvegicus,* was recovered for laboratory testing. CPXV was isolated from brain samples from the rat, and a swab was taken from its paw. Sequence analysis of the rat’s virus indicated complete homology with the girl’s virus. As shown in Figure 2, the sequence analysis clearly demonstrates that the virus was CPXV.

**Figure 2:**
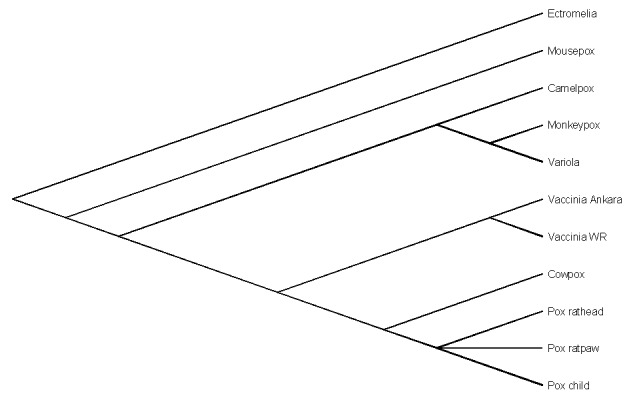
Phylogenetic tree of nucleotide sequences of 163-bp *Orthopoxvirus* fusion gene amplicons from the patient and rat (head and paw), *Cowpox virus*, *Vaccinia virus* (strain Ankara and WR), *Camelpox virus*, *Monkeypox virus*, *Variola virus*, and *Ectromelia virus* (*Mousepox virus*). The nucleotide sequences were aligned by using BioEdit software package (T. Hall, Dept. of Microbiology, Raleigh, NC). Phylogenetic relationships were determined by using the Lasergene software packages (DNASTAR Inc., Madison, WI).

Human CPXV is a rare zoonosis, which is transmitted as a result of contact with animals.

Usually localized at the site of inoculation, CPXV lesions progress from a papule through vesiculation and pustulation into an ulcerative nodule, which is covered with an elevated border with a black eschar. Ulcera heal with scar formation. Differential clinical diagnosis of CPXV includes herpes virus infection, anthrax, and orf (caused by a *Parapoxvirus*). CPXV is almost always a self-limiting disease in immunocompetent hosts.

Although wild rodents are the reservoir hosts of CPXV (1), transmission to humans has only been described from accidental hosts such as infected cats, cows, and animals in zoos and circuses (2–4). Circumstantial evidence of rodents as source of infection has been reported in two human cases: one infection was associated with a suspected rat bite (5); the other infection was in a person who cared for a sick wild field mouse (6). Person-to-person transmission has not been reported.

Our case is the first proven wild rodent-to-human transmission. Serologic surveys have shown that CPXV is a widespread endemic infection in European wild rodents with the highest seroprevalence in bank voles (*Clethrionomys glareolus*), wood mice (*Apodemus sylvaticus*) and field voles (*Microtus agretis*) (1). Seroprevalence rates in rats are not available; therefore, whether rats might also be reservoir hosts or act as liaison (accidental) hosts is unclear. Since smallpox has not occurred naturally anywhere in the world since 1977, immunization with *Vaccinia* *virus* has been discontinued, which has led to a declined cohort immunity to orthopoxviruses including CPXV, and thus may result in an increased incidence of human CPXV infections (2,7). Physicians must be aware that zoonosis may not only be contracted from accidental hosts (e.g., cats and cows) but also directly from a primary natural reservoir (rodents).

## References

[1] Chantrey J, Meyer H, Baxby D, Begon M, Bown KJ, Haxel SM, Cowpox: reservoir hosts and geographic range. Epidemiol Infect. 1999;122:455–60. 10.1017/S0950268899002423 10459650 PMC2809641

[2] Willemse A, Egberink HF. Transmission of cowpox virus infection from domestic cat to man. Lancet. 1985;1:1515. 10.1016/S0140-6736(85)92299-8 2861449

[3] Vestey JP, Yirrel DL, Aldridge RD. Cowpox/catpox infection. Br J Dermatol. 1991;124:74–8. 10.1111/j.1365-2133.1991.tb03285.x 1847067

[4] Baxby D, Bennett M, Getty B. Human cowpox 1969–93: a review based on 54 cases. Br J Dermatol. 1994;131:598–607. 10.1111/j.1365-2133.1994.tb04969.x 7999588

[5] Postma BH, Diepersloot RJA, Niessen GJCM, Droog RP. Cowpox-virus-like infection associated with rat bite. Lancet. 1991;337:733–4. 10.1016/0140-6736(91)90317-I 1672197

[6] Lewis-Jones MS, Baxby D, Cefai C, Hart CA. Cowpox can mimic anthrax. Br J Dermatol. 1993;129:625–7. 10.1111/j.1365-2133.1993.tb00499.x 8251366

[7] Vestey JP, Yirrel DL, Aldridge RD. Cowpox/catpox infection. Br J Dermatol. 1991;124:74–8. 10.1111/j.1365-2133.1991.tb03285.x 1847067

